# Fibrate and the risk of cardiovascular disease among moderate chronic kidney disease patients with primary hypertriglyceridemia

**DOI:** 10.3389/fendo.2024.1333553

**Published:** 2024-02-13

**Authors:** Chieh-Li Yen, Pei-Chun Fan, Cheng-Chia Lee, Jia-Jin Chen, Chao-Yu Chen, Yi-Ran Tu, Pao-Hsien Chu, Ching-Chung Hsiao, Yung-Chang Chen, Chih-Hsiang Chang

**Affiliations:** ^1^ Kidney Research Center, Department of Nephrology, Chang Gung Memorial Hospital, Taoyuan, Taiwan; ^2^ College of Medicine, Chang Gung University, Taoyuan, Taiwan; ^3^ Department of Cardiology, Chang Gung Memorial Hospital, Taoyuan, Taiwan

**Keywords:** TG, chronic kidney disease, hypertriglyceridemia, fibrate, AMI, stroke

## Abstract

**Introduction:**

Hypertriglyceridemia is the most prevalent dyslipidemia in patients with chronic kidney disease (CKD). However, research about fibrate treatment in CKD patients is limited, and assessing its benefits becomes challenging due to the frequent concurrent use of statins. Thus, this study is aimed to investigate the role of fibrate in CKD stage 3 patients with hypertriglyceridemia who did not receive other lipid-lowering agents.

**Methods:**

This study enrolled patients newly diagnosed CKD3 with LDL-C<100mg/dL and had never received statin or other lipid-lowering agents from Chang Gung Research Database. The participants were categorized into 2 groups based on the use of fibrate: fibrate group and non-fibrate group (triglyceride >200mg/dL but not receiving fibrate treatment). The inverse probability of treatment weighting was performed to balance baseline characteristics.

**Results:**

Compared with the non-fibrate group (n=2020), the fibrate group (n=705) exhibited significantly lower risks of major adverse cardiac and cerebrovascular events (MACCEs) (10.4% vs. 12.8%, hazard ratios [HRs]: 0.69, 95% confidence interval [CI]: 0.50 to 0.95), AMI (2.3% vs. 3.9%, HR: 0.52, 95% CI: 0.37 to 0.73), and ischemic stroke (6.3% vs. 8.0%, HR: 0.67, 95% CI: 0.52 to 0.85). The risk of all-cause mortality (5.1% vs. 4.5%, HR: 1.09, 95% CI: 0.67 to 1.79) and death from CV (2.8% vs. 2.3%, HR: 1.07, 95% CI: 0.29 to 2.33) did not significantly differ between the 2 groups.

**Conclusion:**

This study suggests that, in moderate CKD patients with hypertriglyceridemia but LDL-C < 100mg/dL who did not take other lipid-lowering agents, fibrates may be beneficial in reducing cardiovascular events.

## Introduction

Chronic kidney disease (CKD) has emerged as a global public health issue (1). Among all complications of CKD, cardiovascular disease (CVD) is the leading cause of death (2, 3), and the risks increase as renal function declines (4). Patients with CKD commonly face mortality attributed to CVD prior to the onset of end stage kidney disease (ESKD) (3). The risk of CVD in patients with CKD is caused by the combination of atherosclerotic cardiovascular disease (ASCVD) and nonatherosclerotic CVD, such as fluid overload related to heart failure and hyperkalemia related to arrythmia (5). Notably, in the early and moderate stages of CKD, ASCVD accounts for the majority of CV events, while the nonatherosclerotic CVD would only be predominant until the pre-ESKD stage (4, 5). Thus, reducing the risk of ASCVD remains critical for most patients with CKD.

To date, reducing low-density lipoprotein cholesterol (LDL-C) levels by using statin is the most effective treatment for lowering the risk of ASCVD in the general population (6, 7). However, while many studies have demonstrated that elevated levels of triglyceride (TG) are associated with an increased risk of ASCVD (8, 9), whether reduction of TG levels with fibrate treatment can effectively reduce the risks of ASCVD remains uncertain. A recent randomized controlled trial (RCT) indicated that combined fibrate and statin treatment provides no additional benefit relative to statin treatment alone (10). An increasing body of evidence demonstrates that fibrate treatment has weaker effects on the general population compared with statin treatment (11, 12). However, for patients with CKD, we believe that the benefit of fibrate treatment should be re-evaluated for 3 reasons. First, although SHARP study has demonstrated that statin treatment can reduce the risk of CV events in patients with CKD (13), the benefits are less apparent than that in the other high-risk populations (14, 15). Thus, other lipid-lowering agents might be required to further reduce the CV risks in CKD patients. Second, CKD is associated with specific quantitative and qualitative changes in various lipid components, which are apparently different from general populations. For example, the levels of LDL-C would remain stable or slightly decrease, the levels of high-density lipoprotein cholesterol (HDL-C) would decrease, and the levels of TG would substantially increase (16, 17). Thus, fibrate treatment, which can reduce TG levels and elevate HDL-C levels, may be more suitable for patients with CKD than for the general population. Third, a previous *post-hoc* analysis of FIELD study and a meta-analysis have found that the protective effect of fenofibrate against CV events is more prominent in a subgroup of patients with moderate CKD compared to non-CKD patients; however, the reliability of their findings was limited by their small sample size (18, 19).

Thus, this large-scale cohort study investigated whether the use of fibrate is associated with a lower risk of ASCVD. Since a previous study suggested that the concurrent use of statin may have confounded its results on the effects of fibrate (12), our study specifically enrolled patients with only hypertriglyceridemia who were not using statins or any other type of lipid-lowering agents to focus on the impact of fibrate treatment.

## Methods

### Data source

This study was performed on the basis of Chang Gung Research Database (CGRD), which covers the Chang Gung Memorial Hospital system. This system includes 4 tertiary medical centers and 3 teaching hospitals across different regions and is the largest medical network of Taiwan, accounting for approximately 10% of all medical services rendered in Taiwan. The CGRD has deidentified medical data pertaining to the patient’s medication prescriptions, outpatient visits, inpatients orders, procedure interventions, laboratory data, and examination reports (20). Data in the CGRD that may be linked to specific patients or medical providers were encrypted and scrambled before use in this study to protect patient privacy. Thus, the requirement for written informed consent was waived. This study was approved by the Institutional Review Board of the Chang Gung Medical Foundation (approval number: 201900840B0).

### Patient selection and study design

**Figure 1:**
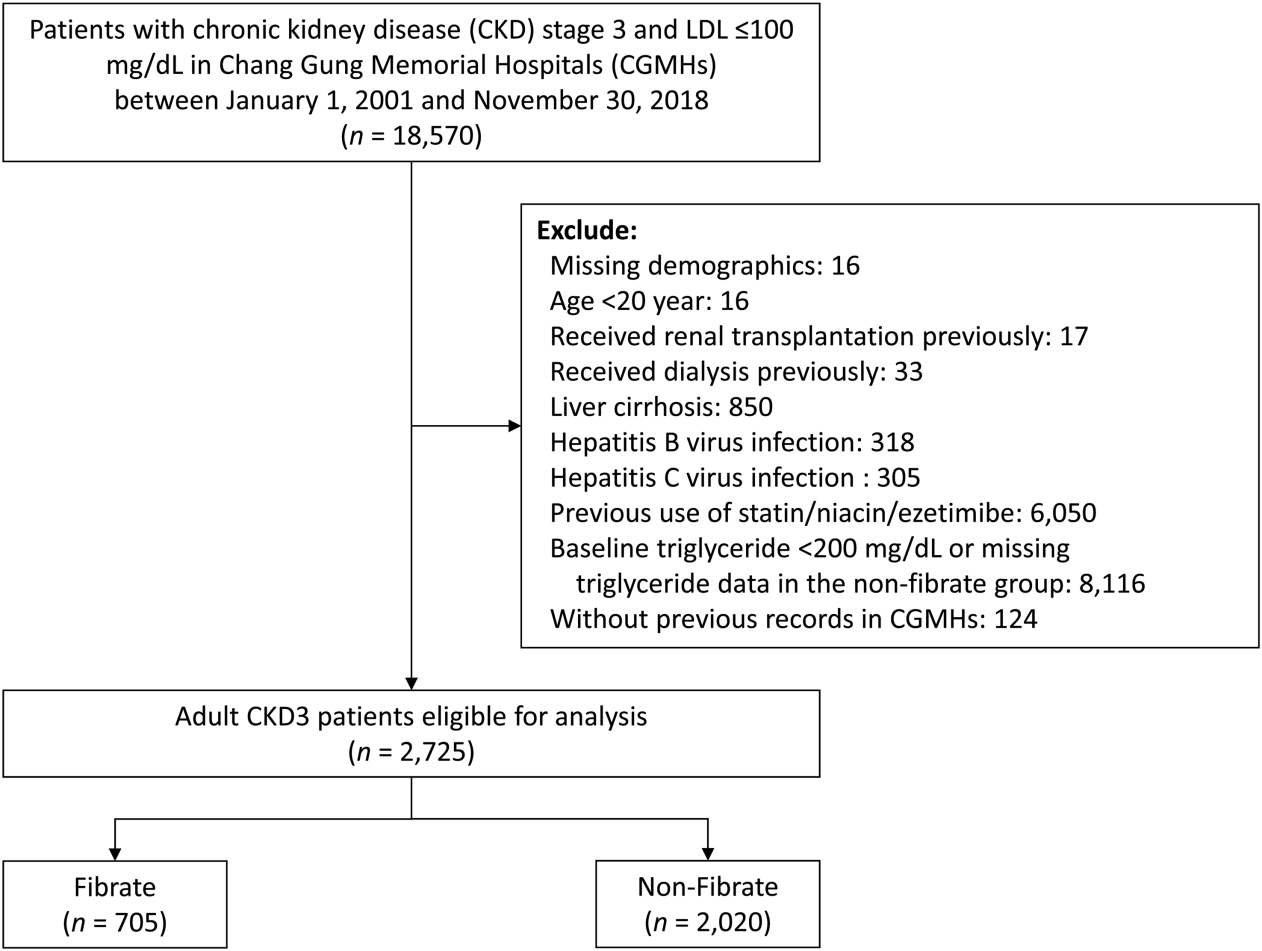
Patient inclusion–exclusion flowchart.

The flow of patient selection is illustrated in **Figure 1**. We identified patients aged >20 years with a diagnosis of stage 3 CKD at any time between 2001 and 2018. In this study, the diagnosis of stage 3 CKD was indicated by 2 consecutive records of estimated glomerular filtration rate (eGFR) between 30 and 60 mL/min/1.73 m^2^ at least 3 months apart, and the second date of eGFR was defined as the index date. To focus on primary hypertriglyceridemia, patients with LDL-C levels exceeding 100mg/dL or those undergoing statin treatment were excluded. In addition, to control for confounders, patients with normal TG levels (TG <200 mg/dL) in the non-fibrate group were excluded. The following patients were excluded: (1) patients with missing data on demographic characteristics; (2) patients undergoing kidney transplantations or any type of dialysis before the index date; (3) patients with liver dysfunction, including hepatitis, virus infection, or liver cirrhosis; and (4) patients treated with other types of lipid-lowering agents, including ezetimibe and niacin. The eligible patients with stage 3 CKD not treated with statin were categorized into 2 study groups: a fibrate group comprising patients treated with fibrate, and a non-fibrate group comprising patients not treated with fibrate. Patients were assigned to either group according to their prescription (fibrate or non-fibrate) within 3 months prior to the index date.

### Covariates

The following demographic and clinical characteristics were adjusted for: age, sex, body mass index [BMI], the primary renal diseases, comorbidities, medications, laboratory examination results at baseline, and the number of admissions in the previous year before the index date. The comorbidities were hypertension, diabetes mellitus (DM), atrial fibrillation, peripheral artery disease, dementia, heart failure, myocardial infarction, and stroke. The ICD-9-CM and ICD-10-CM codes for comorbidities in this study were listed in **Supplementary Table 1**. The primary renal diseases were hypertensive nephropathy, DM nephropathy, chronic glomerulonephritis (eg, lupus nephritis, IGA nephropathy, and focal segmental glomerulosclerosis), and other forms of renal disease (eg, obstructive nephropathy and interstitial nephritis). Comorbidities and primary disease for CKD was indicated by the presence of at least 2 outpatient visits or 1 inpatient stay reported in the year prior to the index date. Baseline laboratory examination results, including the estimated glomerular filtration rate (eGFR) and levels of glycohemoglobin (HbA1c), proteinuria, serum creatinine, blood urine nitrogen, uric acid, sodium, potassium, and hemoglobin were obtained using the most recent record within 3 months preceding the index date. To avoid lipid profile data obtained prior to the initiation of fibrate treatment, the latest lipid profile data during the first 3-month after the index date were used; these data pertained to the levels of triglyceride, LDL-C, HDL-C, and total cholesterol. Finally, concomitant medications were identified on the basis of prescriptions within 90 days prior to the index date.

### Outcomes definition

The primary outcome of this study was MACCEs, defined as a composite of AMI, CV death, and ischemic stroke. The secondary outcomes were all-cause mortality. The outcomes were identified according to the medical records of the CGRD. All-cause mortality and CV death were identified based on documented mortality records in the CGRD. The occurrence of AMI and ischemic stroke was ascertained in the inpatients setting. The follow-up duration extended from the index date to the date of death, the first occurrence of any study outcome independently, the 5^th^ year of follow-up or until the end date of the study period (November 31, 2018), whichever came first.

### Statistical analysis

There was substantial difference of the baseline characteristics between the study groups (fibrate vs. non-fibrate), which may induce selection bias. Therefore, we created an adjusted cohort using inverse probability treatment weighting (IPTW) with average treatment effect based on the propensity score to balance the baseline data between the two groups. We estimated the propensity score using the generalized boosted model (GBM) with 100,000 regression trees, in which it is known the performance of propensity score analysis is better in machine learning approaches than in conventional methods (e.g., logistic regression) (21). All of the baseline data (as listed in **Table 1**) were included in the calculation of the propensity score, with the exception of the follow up duration, which was replaced with the index date. The balance between groups before and after IPTW was assessed using standardized difference (STD), and an absolute value of <0.2 indicated a non-substantial difference between the groups. Single imputation using an expectation–maximization algorithm was employed to account for the substantial number of missing values in the continuous baseline data. All outcome comparisons were made in the complete imputed data and IPTW-adjusted cohort.

**Table 1:** Baseline characteristics of the patients with and without use of fibrate.

Variable	Before IPTW*					After IPTW#		
	Available number	Total (*n* = 2,725)	Fibrate (*n* = 705)	Non-Fibrate (*n* = 2,020)	STD	Fibrate	Non-Fibrate	STD
Demographics								
Age, years	2,725	63.6 ± 12.0	61.9 ± 11.4	64.2 ± 12.1	-0.20	62.7 ± 11.3	63.7 ± 12.1	-0.09
Age ≥65 years	2,725	1,339 (49.1)	298 (42.3)	1,041 (51.5)	-0.19	44.7	49.6	-0.10
Male	2,725	1,704 (62.5)	455 (64.5)	1,249 (61.8)	0.06	64.4	62.5	0.04
Body mass index, kg/m^2^	2,054	26.6 ± 4.9	26.6 ± 4.6	26.6 ± 5.0	<0.01	26.7 ± 4.0	26.7 ± 4.4	<0.01
Primary renal disease	2,725							
Hypertension nephropathy		657 (24.1)	135 (19.1)	522 (25.8)	-0.16	21.9	24.7	-0.07
Diabetes nephropathy		1,507 (55.3)	464 (65.8)	1,043 (51.6)	0.29	60.3	53.3	0.14
Chronic glomerulonephritis		276 (10.1)	64 (9.1)	212 (10.5)	-0.05	10.5	10.3	0.01
Others$		285 (10.5)	42 (6.0)	243 (12.0)	-0.21	7.2	11.7	-0.15
Comorbidities								
Hypertension	2,725	1,959 (71.9)	546 (77.4)	1,413 (70.0)	0.17	74.0	70.2	0.08
Diabetes mellitus	2,725	1,658 (60.8)	502 (71.2)	1,156 (57.2)	0.29	65.0	58.7	0.13
Atrial fibrillation	2,725	111 (4.1)	33 (4.7)	78 (3.9)	0.04	4.6	3.7	0.04
Peripheral artery disease	2,725	89 (3.3)	23 (3.3)	66 (3.3)	<0.01	3.3	3.3	<0.01
Dementia	2,725	73 (2.7)	21 (3.0)	52 (2.6)	0.02	3.1	2.5	0.04
Heart failure	2,725	103 (3.8)	31 (4.4)	72 (3.6)	0.04	3.4	3.5	<0.01
Myocardial infarction	2,725	84 (3.1)	32 (4.5)	52 (2.6)	0.11	3.5	2.6	0.05
Stroke	2,725	215 (7.9)	78 (11.1)	137 (6.8)	0.15	8.2	7.1	0.04
Number of admissions in the previous year	2,725							
0		1,967 (72.2)	541 (76.7)	1,426 (70.6)	0.14	75.9	71.0	0.11
1-2		731 (26.8)	157 (22.3)	574 (28.4)	-0.14	23.1	28.0	-0.11
≥3		27 (1.0)	7 (1.0)	20 (1.0)	<0.01	1.0	1.0	0.01
Medication at baseline								
ACEi/ARB	2,725	1,029 (37.8)	320 (45.4)	709 (35.1)	0.21	43.1	36.9	0.13
Beta-blockers	2,725	509 (18.7)	142 (20.1)	367 (18.2)	0.05	20.1	18.9	0.03
Calcium-channel blocker	2,725	671 (24.6)	178 (25.2)	493 (24.4)	0.02	24.7	24.9	-0.01
Spironolactone	2,725	67 (2.5)	13 (1.8)	54 (2.7)	-0.06	1.3	2.6	-0.10
Loop diuretics	2,725	205 (7.5)	39 (5.5)	166 (8.2)	-0.11	4.7	7.9	-0.13
Nitrates	2,725	243 (8.9)	65 (9.2)	178 (8.8)	0.01	8.3	9.0	-0.02
Vasodilator	2,725	29 (1.1)	6 (0.9)	23 (1.1)	-0.03	0.6	1.1	-0.05
Thiazide	2,725	163 (6.0)	50 (7.1)	113 (5.6)	0.06	6.6	5.8	0.03
NSAIDs	2,725	77 (2.8)	19 (2.7)	58 (2.9)	-0.01	3.2	2.9	0.02
Steroid	2,725	20 (0.7)	4 (0.6)	16 (0.8)	-0.03	0.4	0.8	-0.05
Proton pump inhibitor	2,725	126 (4.6)	21 (3.0)	105 (5.2)	-0.11	4.0	5.2	-0.06
Insulin	2,725	163 (6.0)	29 (4.1)	134 (6.6)	-0.11	4.4	6.7	-0.10
OHAs	2,725	870 (31.9)	332 (47.1)	538 (26.6)	0.43	40.4	29.3	0.23
Pentoxifylline	2,725	76 (2.8)	15 (2.1)	61 (3.0)	-0.06	3.0	3.1	<0.01
Sodium bicarbonate	2,725	9 (0.33)	2 (0.28)	7 (0.35)	-0.01	0.11	0.35	-0.05
Laboratory data at baseline								
Creatinine, mg/dL	2,725	1.41 ± 0.29	1.38 ± 0.27	1.41 ± 0.30	-0.11	1.40 ± 0.28	1.41 ± 0.29	-0.04
eGFR, ml/min/1.73m^2^	2,725	47.5 ± 8.3	48.8 ± 7.9	47.1 ± 8.4	0.21	48.1 ± 8.3	47.3 ± 8.4	0.10
Blood urine nitrogen, mg/dL	1,438	24.0 ± 11.4	23.0 ± 9.3	24.2 ± 11.8	-0.12	22.6 ± 7.5	23.3 ± 9.2	-0.08
Proteinuria group, mg/dL	1,518							
Negative or Trace (0-29)		922 (60.7)	227 (68.0)	695 (58.7)	0.19	63.7	59.1	0.09
1+ or 2+ (30-299)		413 (27.2)	66 (19.8)	347 (29.3)	-0.22	23.0	28.5	-0.13
3+ or 4+ (≥300)		183 (12.1)	41 (12.3)	142 (12.0)	0.01	13.3	12.4	0.03
Potassium, mg/dL	1,666	4.1 ± 0.6	4.2 ± 0.5	4.1 ± 0.6	0.23	4.2 ± 0.4	4.2 ± 0.5	0.12
Sodium, mg/dL	1,325	138.6 ± 5.0	138.8 ± 4.5	138.6 ± 5.1	0.05	139.1 ± 3.2	138.9 ± 3.7	0.07
HbA1C,	1,917	7.9 ± 2.2	7.8 ± 1.8	8.0 ± 2.3	-0.13	7.6 ± 1.7	7.8 ± 2.0	-0.09
Albumin, g/dL	873	3.8 ± 0.7	4.0 ± 0.7	3.7 ± 0.8	0.37	3.9 ± 0.5	3.9 ± 0.5	0.16
Hemoglobin, g/dL	1,587	12.5 ± 2.2	12.6 ± 2.1	12.5 ± 2.2	0.03	12.8 ± 1.7	12.7 ± 1.9	0.05
Uric acid, mg/dL	1,632	7.6 ± 2.1	6.7 ± 2.0	7.8 ± 2.1	-0.58	7.2 ± 1.5	7.6 ± 1.7	-0.26
Lipids profile during the 3-month follow up								
LDL, mg/dL	2,725	77.3 ± 17.2	78.9 ± 16.5	76.7 ± 17.4	0.13	78.0 ± 16.6	76.7 ± 17.4	0.08
HDL, mg/dL	2,142	36.1 ± 10.4	38.4 ± 11.2	35.3 ± 10.0	0.29	36.8 ± 9.3	35.4 ± 9.1	0.15
Total cholesterol, mg/dL	2,249	176.5 ± 43.2	173.4 ± 49.1	177.6 ± 41.0	-0.09	175.9 ± 40.5	177.4 ± 39.3	-0.04
Triglyceride, mg/dL‡	2,719	336.6 ± 299.3	313.0 ± 381.7	344.8 ± 264.5	-0.10	332.0 ± 295.9	351.4 ± 269.0	-0.07
Follow-up duration, year	2,725	7.2 ± 4.5	7.5 ± 4.1	7.1 ± 4.7	0.09	7.3 ± 4.0	7.1 ± 4.6	0.04

STD, standardized difference; IPTW, inverse probability of treatment weighting; ACEi/ARB, angiotensin-converting enzyme inhibitors/angiotensin receptor blocker; NSAIDs, non-steroidal anti-inflammatory drugs; OHAs, oral hypoglycemic agents; eGFR, estimated glomerular filtration rate; HbA1C, glycated hemoglobin; LDL, low-density lipoprotein; HDL, high-density lipoprotein.

* Data were presented as frequency (percentage) or mean ± standard deviation.

Data were presented as percentage or mean ± standard deviation.

$ Including interstitial nephritis, obstructive nephropathy, polycystic kidney and others.

‡ There were six patients with missing data of baseline triglyceride levels in the fibrate group.

The risk of fatal outcomes between the fibrate and non-fibrate groups was compared using the Cox proportional hazard model. The incidence of other time-to-event outcomes between groups was compared using the Fine and Gray subdistribution hazard model which considers all-cause mortality during follow-up as a competing risk. Further, subgroup analysis was conducted on MACCEs stratified by the subgroup variables: age (<65 vs. ≥65 years), sex, hypertension, DM and CKD stage (3a vs. 3b). At last, we compared the risk of MACCEs in patients who took fibrate and in patients who did not take fibrate with different triglyceride levels and HDL-C levels. The analysis was adjusted for all of the covariates (listed in **Table 1**). Due to the existence of potential imbalance in some covariates (i.e., uric acid, use of oral hypoglycemic agents [OHAs] and antiplatelet agents; absolute STD values of >0.2) between groups even after IPTW, additional adjustments were made in the above mentioned regression models. Analyses were performed using SAS software, Version 9.4 (SAS Institute, Cary, NC, USA). A two-sided *P* value of <0.05 was considered significant.

## Results

### Patient characteristics

As illustrated in **Figure 1**, we initially enrolled 2725 adult patients with a diagnosis of stage 3 CKD with LDL <100 mg/dL who had never received statin or other lipid-lowering treatment. Of these patients, 705 patients were assigned to the fibrate group according to the prescription of fibrate within 3 months preceding the index date, and 2020 patients were assigned to the non-fibrate group because they had TG level > 200 mg/dL and did not receive fibrate treatment. In fibrate group, 65.3% of patients received fenofibrate, while 34.7% received gemfibrozil. The patient demographic and clinical characteristics are shown in **Table 1**. The mean eGFRs for the fibrate group and the non-fibrate group were 48.8 and 47.1 ml/min/1.73 m^2^, respectively. The mean TG levels for the fibrate group and the non-fibrate group were 313.0 and 344.8 mg/dL, respectively. In the fibrate group, 46% of patients had follow-up TG levels below 200 mg/dL, while the remaining 54% had follow-up TG levels exceeding 200 mg/dL. The mean LDL-C levels for the fibrate group and the non-fibrate group were 78.9 and 76.7 mg/dL, respectively; and the mean HDL-C levels for the fibrate group and the non-fibrate group were 38.4 and 35.3 mg/dL, respectively. We balanced the covariates between the groups by using IPTW. Before IPTW, the fibrate group was younger; had a higher prevalence of DM and DM nephropathy and lower prevalence of lower proteinuria; and had more prescriptions of angiotensin converting enzyme inhibitors/angiotensin II receptor blockers, anti-platelet agents, and oral OHA compared with those of the non-fibrate group (the absolute standard deviation [STD] values >0.2). The follow-up periods (until the date of death or the end of study period) for the fibrate group and the non-fibrate group were 7.5 and 7.1 years, respectively. After IPTW, except for the prescriptions of OHA, all other absolute STD values were less than 0.2, and most were less than 0.1, which indicates covariate balance (**Table 1**).

### Five-year Follow-up outcomes

**Figure 2:**
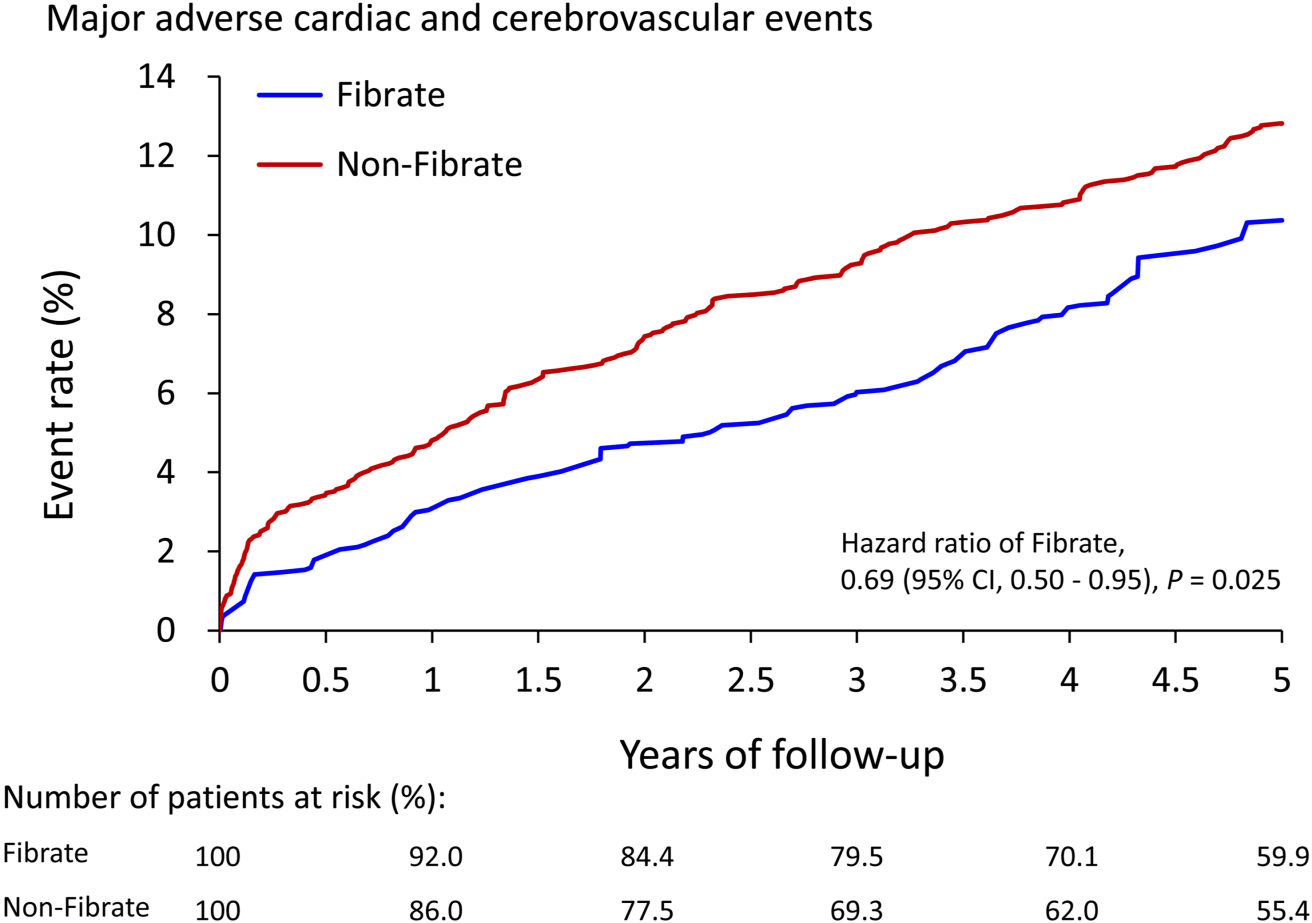
The cumulative event rate of major adverse cardiac and cerebrovascular events of patients with and without use of fibrate in the IPTW-adjusted cohort. IPTW, inverse probability of treatment weighting; CI, confidence interval.

As presented in **Table 2**, after 5-year follow-up, the fibrate group exhibited a significantly lower incidence of MACCE (10.4% vs. 12.8%, hazard ratios [HRs]: 0.69, 95% confidence interval [CI]: 0.50 to 0.95), AMI (2.3% vs. 3.9%, HR: 0.52, 95% CI: 0.37 to 0.73), and ischemic stroke (6.3% vs. 8.0%, HR: 0.67, 95% CI: 0.52 to 0.85) compared with the non-fibrate group. The risk of all-cause mortality (5.1% vs. 4.5%, HR: 1.09, 95% CI: 0.67 to 1.79) and death from CV (2.8% vs. 2.3%, HR: 1.07, 95% CI: 0.29 to 2.33) did not significantly differ between the 2 groups. **Figure 2** presents the cumulative event rates of MACCE.

**Table 2:** Time to event outcomes during 5 years follow up.

Outcome	Before IPTW*		After IPTW#			
	Fibrate (*n* = 705)	Non-Fibrate (*n* = 2,020)	Fibrate	Non-Fibrate	HR/SHR (95% CI) of Fibrate	*P* value
All-cause death	33 (4.7)	96 (4.8)	5.1	4.5	1.09 (0.67–1.79)	0.723
MACCEs						
Cardiovascular death	16 (2.3)	49 (2.4)	2.8	2.3	1.07 (0.52–2.19)	0.858
Acute myocardial infarction	16 (2.3)	80 (4.0)	2.3	3.9	0.52 (0.37–0.73)	<0.001
Ischemic stroke	49 (7.0)	159 (7.9)	6.3	8.0	0.67 (0.52–0.85)	<0.001
Composite outcome$	76 (10.8)	259 (12.8)	10.4	12.8	0.69 (0.50–0.95)	0.025

IPTW, inverse probability of treatment weighting; HR, hazard ratio; SHR, subdistribution hazard ratio; CI, confidence interval; MACCEs, major adverse cardiac and cerebrovascular events.

* Data were presented as frequency (percentage).

Data were presented as percentage.

$ Any of cardiovascular death, acute myocardial infarction or ischemic stroke.

### Subgroup analysis

**Figure 3:**
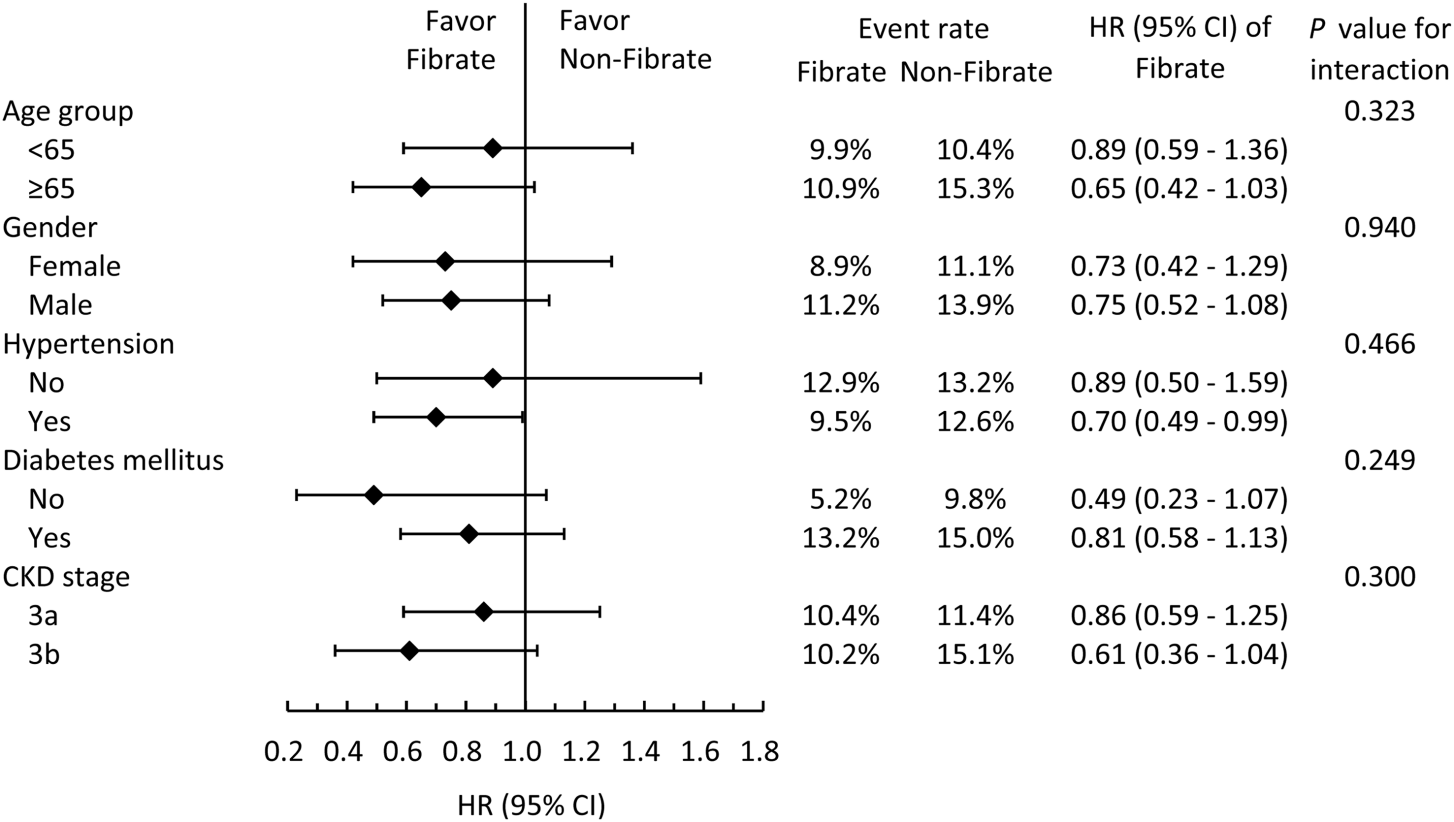
Subgroup analysis of major adverse cardiac and cerebrovascular events stratified by pre-specified baseline characteristics in the IPTW adjusted cohort. IPTW, inverse probability of treatment weighting; HR, hazard ratio; CI, confidence interval; CKD, chronic kidney disease.

To analyze whether clinical conditions modified the association between the use of fibrate and primary outcomes, we performed subgroup analyses for MACCE (**Figure 3**). The results demonstrated that the beneficial effects of fibrate persisted regardless of whether one of many clinical conditions were present or absent, and all interaction effects were nonsignificant.

**Figure 4:**
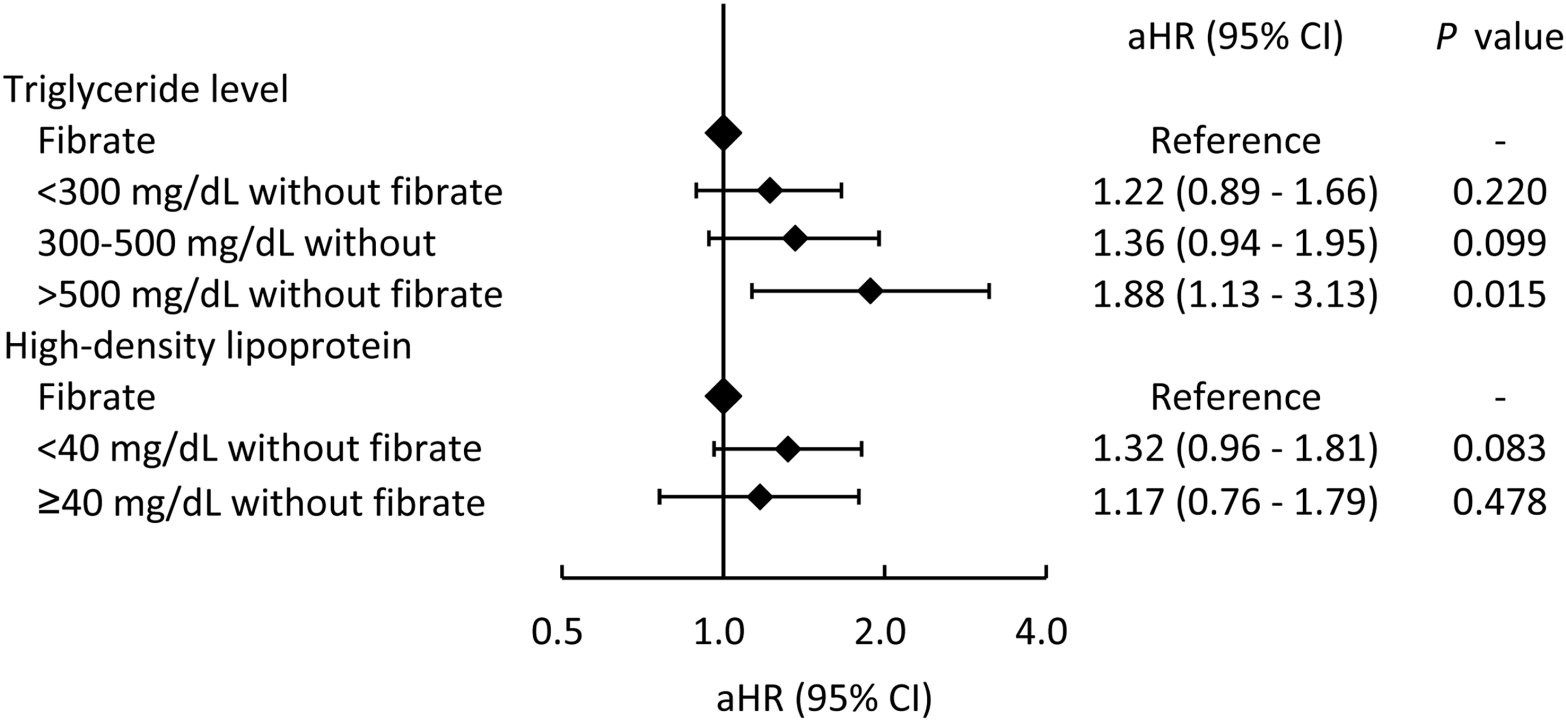
The risks of major adverse cardiac and cerebrovascular events across different triglyceride and high-density lipoprotein levels in patients who did not took fibrate compared those who took fibrate (the reference category). aHR, adjusted hazard ratio; CI, confidence interval.

In addition, to evaluate whether the use of fibrate is more strongly associated with a lower risk of MACCE compared to patients with higher TG levels or with lower HDL-C levels, the fibrate group was taken as the reference group (**Figure 4**). As shown in **Figure 4**, MACCE risk was positively associated with TG level and negatively associated with HDL-C level. In particular, patients in the non-fibrate group with TG > 500mg/dL exhibited a significantly higher risk of MACCE compared with patients in the fibrate group.

On the other hand, we also analyzed the influence of different types of fibrates and the various follow-up TG levels in fibrate-group (**Supplementary Figure 1**). Taking the non-fibrate group as the reference group, patients treated with fenofibrate appeared to experience more pronounced cardiovascular protection compared to patients treated with gemfibrozil, and patients with lower TG levels in the fibrate group demonstrated more substantial cardiovascular protection.

## Discussion

Fibrate, a peroxisome proliferator-activated receptor alpha agonist, effectively reduces TG levels and increases HDL-C levels (12). In addition, a previous study also found that fibrate and statin treatments had equal effects on reducing the ratio of small dense levels of LDL-C (22), which are relatively more atherosclerotic and typically increase as renal function declines (23, 24). However, most previous large-scale RCTs on the effects of fibrates have demonstrated negative results or only mild protective effects against CV events (12). In additional studies, the combination of fibrate and statin treatment could not provide additional benefits in outcomes of CV compared with those of statin treatment alone (10, 25). However, in patients with CKD, because of changes in physical metabolism, hypertriglyceridemia and low levels of HDL-C become the hallmarks of dyslipidemia, and the major change in the levels of LDL-C is qualitative rather than quantitative. The changes associated with dyslipidemia may allow fibrate to perform better in patients with CKD.

This study’s results may differ from those of previous studies due to differences in research design. Furthermore, because the ratio of patients with pure hypertriglyceridemia would increase in patients with CKD and because fibrate plus statin treatment in previous studies have failed, in this study, we only enrolled patients with CKD with LDL-C levels < 100 mg/dL (mean LDL-C: 77.3 mg/dL) not treated with statin or any other lipid-lowering agents. In addition, in the non-fibrate group, this study enrolled patients with TG levels higher than 200 mg/dL to control for confounders. The sample size of this study was relatively large, with a total of 2725 patients with stage 3 CKD with pure hypertriglyceridemia (fibrate group: 705 vs. non-fibrate group: 2020). In contrast, a previous meta-analysis on the effects of fibrate in patients with CKD included only 919 patients with stage 3 CKD (495 in fibrate group vs. 424 in the placebo group) (19). The larger sample size enabled us to conduct additional subgroup analyses.

Regarding MACCEs, the fibrate group had significantly lower risks of AMI and ischemic stroke. These findings remained the same in a further subgroup analysis by sex and age, and comorbidities. In addition, previous studies have found that the use of fibrate in the subgroup with higher TG levels and lower HDL-C levels substantially reduced the risk of CV. This finding is consistent with that of our study that the benefit of fibrate is more obvious in patients with higher TG levels or lower HDL-C levels (26, 27), and patients with lower follow-up TG levels after fibrate treatment demonstrated more substantial cardiovascular protection as well. Thus, in patients with stage 3 CKD, the reduction of the levels of TG and the increase of the levels of HDL-C may reduce the risk of CV events. Notable, the subgroup analysis indicated that the beneficial effect of fibrate is mildly enhanced with the progression of CKD. In a previous *post-hoc* analysis, in which patients were divided according to their renal function, revealed that fibrate treatment substantially reduced the risk of CV in the group with an eGFR of 30 to 59 ml/min/1.73 m^2^ (18). Previous findings and our findings indicate that the protective effect of fibrate against CV events in patients with CKD warrants further investigation. Finally, this study demonstrated that fenofibrate appears to offer more pronounced cardiovascular protection than gemfibrozil. However, further research is warranted to validate these findings.

While an observational study cannot definitively elucidate the underlying mechanisms, we can still propose several potential pathways to explain the benefits of fibrates in CKD patients. One potential mechanism involves the role of small dense LDL-C (sdLDL-C), which is known to increase with CKD progression and is more atherogenic than regular LDL-C (28, 29). Previous research has also shown that fibrates could reduce the ratio of sdLDL-C (22, 30), which may partially account for the protective effect observed in CKD patients. Another possible mechanism to account for the results of our study is the impact on the catabolic rate of apolipoprotein B. Apolipoprotein B plays a fundamental role in the atherosclerotic process (31, 32), and in CKD patients, the catabolic rates of VLDL-apolipoprotein B are known to decrease (32). Prior research has demonstrated that fibrates can increase the catabolic rate of VLDL-apolipoprotein B, which might also contribute to the cardiovascular benefits associated with fibrates (33, 34). Further research, incorporating more detailed lipid profiles, including sdLDL-C levels and apolipoprotein B, in CKD patients, is necessary to validate our speculations.

This study has several limitations. First, the observational study design of this study might have inherent bias that could not be simply eliminated by using propensity score weighting. Second, this study used data from a database, which does not guarantee the long-term compliance of fibrate. Third, because we intended to include more patients into the fibrate group, we did not exclude patients with TG levels >200 mg/dL in the fibrate group. Thus, the difference in mean TG levels between the fibrate group and non-fibrate group was only 30 mg/dL. However, theoretically, this limitation may be disadvantageous to fibrate group. Furthermore, we conducted an additional analysis of the CV outcomes within fibrate group, stratified by different TG levels, and demonstrated that patients with lower TG levels in the fibrate group had more substantial cardiovascular protection. Forth, the lack of laboratory results during the study and the lack of information regarding the dosage of fibrate represent two crucial weakness is this study. Finally, this study only enrolled patients with pure hypertriglyceridemia and excluded patients treated with statin. Therefore, our research team is designing further study to verify whether the use of fibrate can reduce the risk of CV events in CKD patients already under statin treatment.

In conclusion, by simplifying the research target to patients with stage 3 CKD with pure hypertriglyceridemia, this study demonstrated that the use of fibrate could reduce the risks of MACCE, such as AMI and ischemic stroke, in the target population. This study provides evidence that fibrate can be prescribed for treating pure hypertriglyceridemia in patients with stage 3 CKD without undergoing statin treatment. Further large-scale RCTs or cohort studies on the corresponding effect are warranted.

## Data availability statement

The original contributions presented in the study are included in the article/**Supplementary Material**. Further inquiries can be directed to the corresponding author.

## Ethics statement

The studies involving humans were approved by Board of the Chang Gung Medical Foundation (approval number: 201900840B0). The studies were conducted in accordance with the local legislation and institutional requirements. Written informed consent for participation was not required from the participants or the participants’ legal guardians/next of kin in accordance with the national legislation and institutional requirements.

## Author contributions

CY: Conceptualization, Investigation, Writing – original draft. PF: Data curation, Resources, Writing – review & editing. CL: Conceptualization, Investigation, Writing – review & editing. JC: Formal analysis, Methodology, Writing – review & editing. CC: Data curation, Software, Writing – review & editing. YT: Data curation, Methodology, Writing – review & editing. PC: Project administration, Resources, Supervision, Writing – review & editing. CH: Formal analysis, Methodology, Writing – review & editing. YC: Conceptualization, Project administration, Resources, Writing – review & editing. CC: Conceptualization, Funding acquisition, Writing – review & editing.

## Supplementary material

Supplementary Figure 1 The risks of major adverse cardiac and cerebrovascular events across different triglyceride and different types of fibrate in patients who took fibrate compared those who did not take fibrate (the reference category). aHR, adjusted hazard ratio; CI, confidence interval.
