# Supplementary figures and images for: Fibrate and the risk of cardiovascular disease among moderate chronic kidney disease patients with primary hypertriglyceridemia
Source: Front Endocrinol (Lausanne). 2024 Feb 13;15:1333553. doi: 10.3389/fendo.2024.1333553 (PMC10897040; doi:10.3389/fendo.2024.1333553)

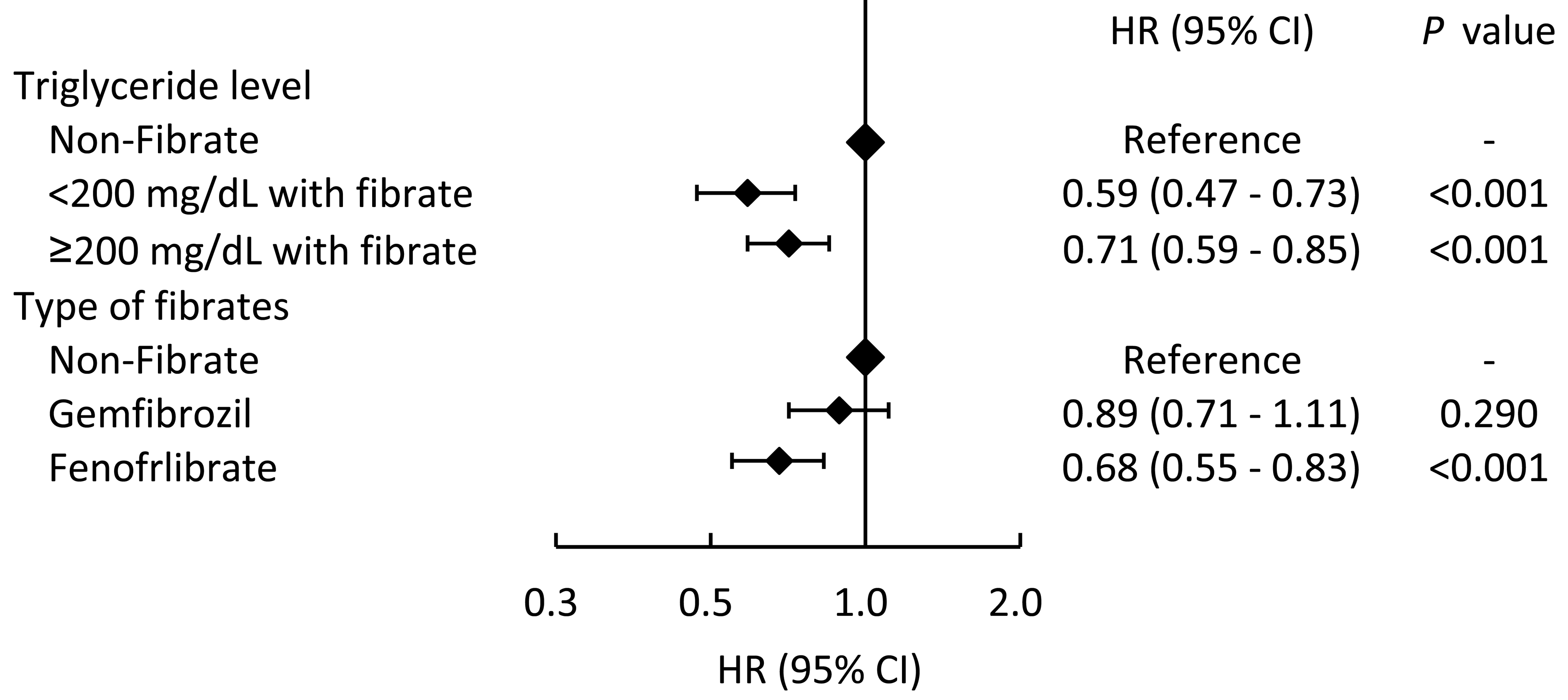

Supplement: Supplementary Figure 1 — The risks of major adverse cardiac and cerebrovascular events across different triglyceride and different types of fibrate in patients who took fibrate compared those who did not take fibrate (the reference category). aHR, adjusted hazard ratio; CI, confidence interval. [file Image_1.tif]
