# Supplementary material for: Fibrate and the risk of cardiovascular disease among moderate chronic kidney disease patients with primary hypertriglyceridemia
Source: Front Endocrinol (Lausanne). 2024 Feb 13;15:1333553. doi: 10.3389/fendo.2024.1333553 (PMC10897040; doi:10.3389/fendo.2024.1333553)
Supplement: Supplementary file 2 [file Table_1.docx]

**Supplemental Table 1**. Disease code use in this study

| **Disease** | **ICD-9-CM** | **ICD-10-CM** |
| --- | --- | --- |
| Liver cirrhosis | 571.2x, 571.5x, 571.6x | K70.30, K74.0, K74.60, K74.69, K74.3, K74.4, K74.5 |
| Hepatitis B virus infection | 070.20, 070.22, 070.30, 070.32, V02.61 | B18.0, B18.1, B16.2, B19.11, B16.9, B19.10 |
| Hepatitis C virus infection | 070.41, 070.44, 070.51, 070.54, 070.70, 070.71, V02.62 | B18.2, B17.10, B17.11, B19.20, B19.21 |
| Hypertension nephropathy | 403.9x | I12 |
| Diabetes nephropathy | 250.4x | E08.2, E09.2, E10.2, E11.2, E13.2 |
| Chronic glomerulonephritis | 582.x | N03 |
| Interstitial nephritis | 583.89 | N11.8, N11.9, N14, N15.8 , N15.9 |
| Obstructive nephropathy | 599.6 | N13 combined with N18.3 to N18.6 |
| Polycystic kidney disease | 753.12, 753.13, 753.14 | Q61.1, Q61.2, Q61.3 |
| Hypertension | 401.xx-405.xx | I10-I15, N262 |
| Diabetes mellitus | 250.xx | E08-E13 |
| Atrial fibrillation | 427.3x | I48 |
| Peripheral arterial disease | 440.xx, 441.xx, 443.xx, 444.0x, 444.8x, 447.8x, 447.9x, 093.0, 437.3, 444.22, 447.1, 557.1, 557.9, V434 | I70, I71, I73, I75, I771, I790, I791, I792, I773, I779, I798, K551, K558, K559, Z958, Z959, I743, I744, I745, I748, I740, I7789 |
| Dementia | 290.xx, 294.xx | F03.90, F05, F01.50, F01.51, F04, F02.80, F02.81, F03.90, F03.91, F06.0, F06.1, F06.8, F09 |
| Heart failure | 428.xx | I50 |
| Myocardial infarction | 410.xx, 412.xx | I21-I22 |
| Stroke | 430.xx–437.xx | I60-I62, I66, I65.1, I65.0, I65.8, I65.9, I63.6, I63.8, I63.9, G45.0, G45.8, G45.1, G45.2, G46.0, G46.1, G46.2, G45.9, G45.4, G46.3, G46.4, G46.5, G46.6, G46.7, G46.8, I67.0, I67.1, I67.2, I67.4, I67.5, I67.6, I67.7, I67.9, I68.0, I68.2, I68.8 |
| Acute myocardial infarction | 410.xx | I21 |
| Ischemic stroke | 433.xx–437.xx | I66, I65.1, I65.0, I65.8, I65.9, I63.6, I63.8, I63.9, G45.0, G45.8, G45.1, G45.2, G46.0, G46.1, G46.2, G45.9, G45.4, G46.3, G46.4, G46.5, G46.6, G46.7, G46.8, I67.0, I67.1, I67.2, I67.4, I67.5, I67.6, I67.7, I67.9, I68.0, I68.2, I68.8 |
| Renal transplantation | V42.0 | Z94.0 |
